# Lifestyle Patterns and Weight Status in Spanish Adults: The ANIBES Study

**DOI:** 10.3390/nu9060606

**Published:** 2017-06-14

**Authors:** Carmen Pérez-Rodrigo, Marta Gianzo-Citores, Ángel Gil, Marcela González-Gross, Rosa M. Ortega, Lluis Serra-Majem, Gregorio Varela-Moreiras, Javier Aranceta-Bartrina

**Affiliations:** 1 FIDEC Foundation, University of the Basque Country (UPV/EHU), Gurtubay s/n, 48010 Bilbao, Spain; carmenperezrodrigo@gmail.com; 2 Department of Physiology, Faculty of Medicine and Nursery, University of the Basque Country (UPV/EHU), Bo Sarriena s/n, Leioa, 48940 Bizkaia, Spain; martagnz_2@hotmail.com; 3 Department of Biochemistry and Molecular Biology II, Institute of Nutrition and Food Sciences, Centre of Biomedical Research, University of Granada, Campus de la Salud, Avda. del Conocimiento, Armilla, 18100 Granada, Spain; agil@ugr.es; 4 CIBEROBN, Biomedical Research Networking Center for Physiopathology of Obesity and Nutrition, Carlos III Health Institute, Madrid 28029, Spain; marcela.gonzalez.gross@upm.es (M.G.-G.); lluis.serra@ulpgc.es (L.S.-M.); 5 ImFINE Research Group, Department of Health and Human Performance, Universidad Politécnica de Madrid, C/Martín Fierro 7, 28040 Madrid, Spain; 6 Department of Nutrition, Faculty of Pharmacy, Complutense University of Madrid, Plaza Ramón y Cajal s/n, 28040 Madrid, Spain; rortega@ucm.es; 7 Research Institute of Biomedical and Health Sciences, Faculty of Health Sciences, University of Las Palmas de Gran Canaria, Paseo Blas Cabrera Felipe “Físico” s/n, 35016 Las Palmas de Gran Canaria, Spain; 8 Department of Pharmaceutical and Health Sciences, Faculty of Pharmacy, CEU San Pablo University, Urb. Montepríncipe, Crta. Boadilla Km. 5.3, Boadilla del Monte, 28668 Madrid, Spain; gvarela@ceu.es or gvarela@fen.org.es; 9 Spanish Nutrition Foundation (FEN), C/General Álvarez de Castro 20. 1a pta, 28010 Madrid, Spain; 10 Department of Food Sciences and Physiology, University of Navarra, C/Irunlarrea 1, 31008 Pamplona, Spain

**Keywords:** cluster analysis, dietary patterns, lifestyle patterns, physical activity, sedentary behavior, obesity, adults

## Abstract

Limited knowledge is available on lifestyle patterns in Spanish adults. We investigated dietary patterns and possible meaningful clustering of physical activity, sedentary behavior, sleep time, and smoking in Spanish adults aged 18–64 years and their association with obesity. Analysis was based on a subsample (*n* = 1617) of the cross-sectional ANIBES study in Spain. We performed exploratory factor analysis and subsequent cluster analysis of dietary patterns, physical activity, sedentary behaviors, sleep time, and smoking. Logistic regression analysis was used to explore the association between the cluster solutions and obesity. Factor analysis identified four dietary patterns, “*Traditional DP*”, “*Mediterranean DP*”, “*Snack DP*” and “*Dairy-sweet DP*”. Dietary patterns, physical activity behaviors, sedentary behaviors, sleep time, and smoking in Spanish adults aggregated into three different clusters of lifestyle patterns: “*Mixed diet-physically active-low sedentary lifestyle pattern*”, “*Not poor diet-low physical activity-low sedentary lifestyle pattern*” and “*Poor diet-low physical activity-sedentary lifestyle pattern*”. A higher proportion of people aged 18–30 years was classified into the “*Poor diet-low physical activity-sedentary lifestyle pattern*”. The prevalence odds ratio for obesity in men in the “*Mixed diet-physically active-low sedentary lifestyle pattern*” was significantly lower compared to those in the “*Poor diet-low physical activity-sedentary lifestyle pattern*”. Those behavior patterns are helpful to identify specific issues in population subgroups and inform intervention strategies. The findings in this study underline the importance of designing and implementing interventions that address multiple health risk practices, considering lifestyle patterns and associated determinants.

## 1. Introduction

Overweight and obesity have progressively increased during the last decades and have become a major issue in public health, both in developed and developing economies across the five continents [1,2]. In Spain, recent data report that more than half of adults aged 18–64 years are classified as overweight or obese [3,4]. This is particularly worrying for the negative impact of this condition on health and quality of life [2,5,6]. In fact, the Global Burden of Disease project highlights that high body mass index (BMI) values were among the main risk factors driving most death and disability combined in the country in 2015 [7].

Overweight and obesity result from an imbalance between energy intake and expenditure. The role of diet in obesity is complex. Most research in this area has focused on specific foods and nutrients [8,9]. Nevertheless, the analysis of food patterns is particularly interesting, since foods are usually consumed in combinations and those may have synergistic, antagonistic, or moderating effects [10]. 

In addition, to date, it is acknowledged that joint interactions of multiple variables acting at different levels influence weight gain, such as lifestyles [11,12,13], sleep, including rhythm, duration or quality of sleep [14,15], eating behaviors [16], socioeconomic level, education, and other factors [4,11]. Data-driven methods explore the similarities between different food options in specific population groups [8]. Specifically, cluster analyses include several techniques aimed at grouping together individuals sharing a number of features or similar lifestyles. This classification allows for a better understanding of the influences of behaviors and lifestyles, as well as the potential cumulative effects of an unhealthy combination of those factors on the development of overweight and obesity [16]. Research of dietary patterns and the potential combination of those with other lifestyles can contribute to identifying effective strategies for the prevention of overweight and obesity among adults, as well as its social and health consequences [8]. 

Considering the above, the objectives of this paper are (a) to identify food patterns in the Spanish adult population; (b) to investigate if energy balance-related behaviors tend to assemble into meaningful patterns in Spanish adults; (c) to describe existing relationships between socio-demographical factors and different lifestyle patterns; and, finally; (d) to analyze the potential association of those correlates with excess body weight. 

## 2. Materials and Methods 

The data for this analysis were obtained from the ANIBES study, an observational cross-sectional study conducted on a random sample of the Spanish population aged 9–75 years. The aims and procedures used in ANIBES have been previously reported [17,18]. 

Briefly, the sample design of the ANIBES study was based on the Spanish population census 2012 according to sex, age, residence, and regional population size. A stratified multistep sampling procedure was used, with random selection of addresses in the municipalities and age and sex quotas for individuals within households. Interlocked quotas were established for age in the regions and size of habitat within the region. The sample selection procedure was based on random paths; 128 sampling points were selected.

The final sample of the study consisted of 2009 individuals (1013 men, 50.4%, 996 women, 49.6%). In addition, a boost sample was recruited for the younger groups (9–12 years; 13–17 years, and 18–24 years) in order to ensure at least 200 individuals in each age group. Data for individuals aged 18–64 years were used for this analysis. That age range is meaningful from a risk prevention point of view, since it includes adults in the active working life span, thus, relevant when designing targeted preventive and health promotion strategies, considering different settings, such as workplaces, primary health care, and community-based interventions. In addition, the focus on the 18–64 year-old range was also relevant for comparison with previous studies conducted in Spain [19]. Data were collected between mid-September and mid-November 2013.

The final protocol of the study was approved by the ethical research committee of the Community of Madrid, Spain.

### 2.1. Measurements

#### 2.1.1. Lifestyle Factors

##### Diet

Dietary intake was assessed by means of a face-to-face 24-h diet recall interview assisted by a food picture atlas to estimate portion sizes. In addition participants completed a three-day food record aided by a tablet device (Samsung Galaxy Tab 2 7.0, Samsung Electronics, Suwon, South Korea), two consecutive weekdays and one weekend day, recording all foods and beverages consumed at home and away from home. Processing of food record inputs, coding and data cleaning has been extensively reported elsewhere [17,18]. Energy and nutrient intakes were calculated using new specifically-developed software, (VD-FEN 2.1 software -Dietary Evaluation Programme, Spanish Nutrition Foundation, Madrid, Spain) for the ANIBES study [20]. Consumption of all food and beverages was arranged into 16 food groups, 45 subgroups, and 754 food items, for in-depth analysis, based on the structure of the food composition database considering similarities in nutrient profile (Supplementary Materials Table S1). 

##### Physical Activity

Physical activity data were collected by face-to-face interview using the validated International Physical Activity Questionnaire (IPAQ) [21]. Total minutes per week were computed for moderate to vigorous physical activity based on the IPAQ guidelines for data processing and analyses [22]. Data were cleaned and truncated based on IPAQ guidelines and previous research [23]. Additionally, the total minutes per week of commuting-related physical activity (walking, biking) were computed. IPAQ data was used for this analysis. Z-scores of minutes per week for each type of activity were calculated. 

##### Sleep Duration

Sleep habits included the number of hours slept per night, on average, as reported by each individual. 

##### Smoking

Smoking habits were assessed by different items in the protocol, including “During the past year, how many cigarettes per day did you smoke on average?”

#### 2.1.2. Body Measurements

Anthropometric measurements were taken individually by trained interviewers, following international standard procedures previously tested in two pilot studies [24], reported in detail elsewhere [17,18]. Body mass index (BMI) was calculated as body weight in kilograms divided by the square of body height in meters. Overweight status was defined as BMI ≥ 25; obesity as BMI ≥ 30.

#### 2.1.3. Covariates

##### Education

The education levels were established in accordance with the Spanish educational system.

After preliminary analysis of the distribution of the variable, categories were collapsed and recoded into a three-point scale, as follows: (1) low (less than seven years of education; primary school or less); (2) medium (7–12 years of education; lower to higher secondary education); and (3) high (13 years or more of education; higher vocational, college, and university studies).

##### Geographical Area

Geographical area in the country was collapsed into four different categories: north-northwest region; eastern region; central region and southern region.

### 2.2. Data Cleaning

Detailed data cleaning procedures have been previously described [17,18]. Participants were considered fully eligible after a verified quality check of the input from the tablet device of adequately completed three-day food records. After data cleaning stages, individuals remained in the database if they had successfully completed both face-to-face interviews during fieldwork and had measured weight, height, and waist circumference data. Of the initial sample of 1655 recruited individuals aged 18–64 years, 1617 individuals satisfied the inclusion criteria and had complete data for all of the variables included in this analysis. 

### 2.3. Data Analysis

All statistical tests were performed using IBM SPSS Statistics for Windows, Version 22.0 (IBM Corp., Armonk, NY, USA). Descriptive statistics were computed for each variable.

#### 2.3.1. Dietary Patterns 

Exploratory factor analysis was performed to identify underlying dietary patterns, using the average food weight (g/day) consumed by each individual (three-day food record plus one-day 24-h recall) from 38 food groups as input variables. Food groups were used to further collapse dietary intake data in order to avoid missing data from non-consumers of episodically consumed foods. *Z*-scores for each food group were calculated to prevent the components being dominated by the foods that provide the highest amounts. Bartlett’s test of sphericity and the Kaiser-Meyer-Olkin (KMO) measure of sampling adequacy were used to verify the appropriateness of factor analysis. Factors were also orthogonally rotated (the varimax option) to enhance the difference between loadings, which allowed for easier interpretability.

Factors were retained based on the following criteria: factor eigenvalue > 1.20, identification of a break point in the scree plot, the proportion of variance explained, and factor interpretability [25].

The strength and direction of the associations between patterns and food groups were described through a rotated factor loading matrix. Food groups with factor loadings > 0.30 and communality > 0.20 were retained in the patterns identified. The factor score for each pattern was constructed by summing the observed intakes of the component food items weighted by the factor loading. A high factor score for a given pattern indicated high intake of the foods constituting that food factor, and a low score indicated low intake of those foods.

#### 2.3.2. Lifestyle Patterns

To identify clusters with similar dietary patterns, physical activities, sedentary activities, sleeping habits and smoking a combination of hierarchical and non-hierarchical clustering analysis was used [26]. The variables used had different arithmetic scales; thus, *Z*-scores were calculated to standardize the dataset before clustering, to avoid a greater contribution to the distance of variables having larger ranges than variables with smaller ranges. Univariate and multivariate outliers (>3 SD) were removed. First, hierarchical cluster analysis was performed using Ward’s method, based on squared Euclidian distances. Several possible cluster solutions were identified and compared to inform the next step, considering the coefficients and fusion level. A non-hierarchical k-means clustering procedure was used, specifying the number of clusters identified in the first step, using a random initial seed and 10 iterations in order to further refine the preliminary solution by optimizing the classification. The final cluster solution was selected based on interpretability and the percent of the study population in each cluster. Reliability and stability of the final cluster solution was tested by randomly taking a subsample (50%) of the total sample and repeating the analyses on this subsample. To check agreement, a kappa statistic was calculated between the cluster solutions of the subsample and that of the total sample. 

Pearson’s chi-square tests were used to investigate the differences in cluster distribution by gender, age group, education level, geographical area, and BMI status. One-way ANOVA was used to compare physical activities, sedentary behaviors and sleep time across clusters stratified by gender and age group. General linear models were used to estimate multivariate means for food consumption and dietary pattern scores across clusters adjusted for age and energy intake. Binary logistic regression analysis was used to explore the prevalence odds ratios for obesity and overweight among lifestyle patterns. The models were adjusted for energy intake, sex, age, educational level, and geographical area. Statistical tests were two-tailed with a 5% level of significance.

## 3. Results

### 3.1. Sample Characteristics

After exclusion of outliers and participants with incomplete data, 1617 subjects aged 18–64 years, 781 men and 836 women, were included in the analyses (Table S2). Some 38% of the sample was classified in the BMI range 25–29.9 and 21.6% had BMI values ≥ 30. There was no significant difference between men and women in age distribution, level of education, or geographical area. However, overweight and obesity rates were significantly higher in men than women.

### 3.2. Dietary Patterns

**Figure 1:**
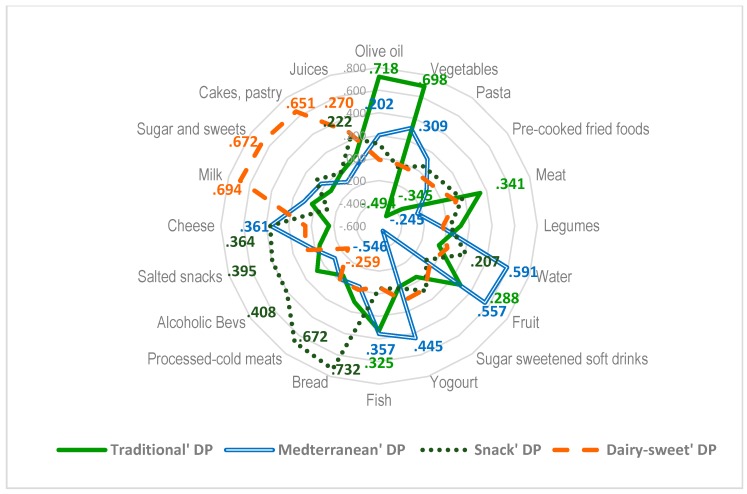
Factor loadings after varimax rotation on identified dietary patterns of food groups retained. Eigenvalues: *Traditional DP* = 2.15; *Mediterranean DP* = 1.64; *Snack DP* = 1.55; *Dairy-sweet DP* = 1.28. Percent of variance explained: *Traditional DP*: 8.74%; *Mediterranean DP:* 8.39%; *Snack DP*: 8.19%; *Dairy sweet* DP: 7.74%. Total variance explained 33.07%. Absolute values less than 0.20 are not shown.

Dietary patterns were computed for the entire sample. Bartlett’s test of sphericity and KMO = 0.591 supported the appropriateness of factor analysis. Four major factors were extracted, which explained 33.1% of the variance in the model. The first dietary pattern (DP) was labeled “*Traditional DP*” which had the highest loading on olive oil and vegetables, high scores on fish, meat, and fruit, and negative scores on pasta and so-called “pre-cooked” foods, which include food items such as croquettes and other processed foods usually prepared deep-fried for consumption. A DP labeled “*Mediterranean DP*” had high scores on water, fruit, yoghourt, fish, vegetables, cheese, and olive oil, and negative scores on meat and sugar sweetened beverages. A DP labeled “*Snack DP*” had high scores on bread, processed and cold meats, alcoholic beverages, salted snacks, cheese, and juices. Finally, “*Dairy-sweet DP*” had high scores on milk, sugar and sweets, cakes, pastry, and juices, and negative scores on alcoholic beverages. Overall, these patterns explained 33.07% of the variance (Figure 1).

Mean factor scores for “*Traditional DP*” and “*Mediterranean DP*” were significantly higher in the oldest age group (50–64 years), while “*Dairy-sweet DP*” factor scores were significantly higher in the younger ones (18–30 years). Men had significantly higher scores than women for “*Snack DP*” adjusted for age and energy intake; “*Mediterranean DP*” and “*Dairy-sweet DP*” had significantly higher scores among women. “*Mediterranean DP*” and “*Traditional DP*” factor scores were significantly higher in people with a higher educational level.

Factor scores for the “*Traditional DP*” and “*Dairy-sweet DP*”, adjusted for energy intake, age, and gender, were significantly higher in the north-northwest region, while “*Mediterranean DP*” and “*Snack DP*” factor scores were significantly higher in the Eastern Mediterranean region. 

### 3.3. Lifestyle Patterns

Based on the four identified DPs, minutes per week of vigorous, moderate physical activity, walking, biking, sedentary time, sleep duration on weekdays, and smoking habits, the three-cluster solution was found to be adequate and meaningful regarding the different patterns. The kappa statistic (κ = 0.94) suggested good agreement. 

Differential characteristics of each cluster are identified by high (above 0) or low *Z*-scores (below 0) comparing cluster centers in *Z*-scores (Figure S1). Cluster 1, labeled “*Mixed diet-physically active-low sedentary lifestyle pattern*”, drew a lifestyle pattern with high scores on the “*Snack DP*” (*Z*-score = 0.42), the “*Mediterranean DP*” (*Z*-score = 0.27), walking (*Z*-score = 0.58), vigorous physical activity (*Z*-score = 2.09) and moderate physical activity (*Z*-score=0.33), combined with low scores on sedentary time (*Z*-score = −0.40) and sleeping (*Z*-score = −0.25). The second cluster, labeled “*Not poor diet-low physical activity-low sedentary lifestyle pattern*”, had low scores on “*Dairy-sweet DP*” (*Z*-score = −0.40), “*Snack DP*” (*Z*-score = −0.16), vigorous physical activity (*Z*-score = −0.34) and sedentary time (*Z*-score = −0.19). Finally, a third cluster labeled “*Poor diet-low physical activity-sedentary lifestyle pattern*” had high scores on “*Dairy-sweet DP*” (*Z*-score = 1.09), “*Snack DP*” (*Z*-score = 0.19) and sedentary time (*Z*-score = 0.73) and scored negatively on moderate (*Z*-score = −0.42) and vigorous physical activity (*Z*-score = −0.26), as well as smoking (*Z*-score = −0.21).

Characteristics of the subjects classified in the lifestyle patterns identified are described in Table 1. The “*Mixed diet-physically active-low sedentary lifestyle pattern*” included 13% of the sample and a significantly higher proportion of men. The “*Not poor diet-low physical activity-low sedentary lifestyle pattern*” included 63.3% of the sample, a significantly higher proportion of women. The “*Poor diet-low physical activity-sedentary lifestyle pattern*” included 23.6% of the sample. 

A higher percentage of people across age groups were classified into the “*Not poor diet-low physical activity-low sedentary lifestyle pattern*”. However, a significantly lower proportion of individuals in the older age group (50–64 years) was classified in the “*Mixed diet-physically active-low sedentary lifestyle pattern*” and a higher proportion of people aged 18–30 years were classified into the “*Poor diet-low physical activity-sedentary lifestyle pattern*”. The highest proportion of people with a lower educational level was classified into the “*Not poor diet-low physical activity-low sedentary lifestyle pattern*”. There was no difference in the clusters by geographical area. 

Prevalence rates of obesity were significantly higher among people allocated in the “*Not poor diet-low physical activity-low sedentary lifestyle pattern*” and overweight in the “*Mixed diet-physically active-low sedentary lifestyle pattern*”, however, those differences were not significant in the stratified analysis by age and gender.

Table 2 describes physical activity behaviors, sedentary, sleep time on weekdays, smoking behavior, and dietary pattern *Z*-scores in the lifestyle patterns. Vigorous physical activity, moderate physical activity, walking time, as well as the *Z*-scores of the Mediterranean DP were significantly higher in men and women classified in the “*Mixed diet-physically active-low sedentary lifestyle pattern*”. *Z*-scores for “*Snack DP*” were also higher in men in that lifestyle pattern. Men and women in the “*Poor diet-low physical activity-sedentary lifestyle pattern*” had significantly higher scores on the “*Dairy-sweet DP*” and sedentary time. 

Consumption of selected food groups and beverages by lifestyle pattern in men and women is described in Table 3. Consumption of fruit, pasta, olive oil, water and alcoholic beverages, particularly wine and beer, was significantly higher in men and women included in the “*Mixed diet-physically active-low sedentary lifestyle pattern*”. Consumption of milk, cakes and pastry, sugar and sweets was significantly higher in men and women classified in the “*Poor diet-low physical activity-sedentary lifestyle pattern*”. Men in this lifestyle pattern showed significantly higher consumption of pre-cooked deep fried foods and high alcoholic content beverages. Women in this pattern had significantly higher consumption of savory snacks, juices and sugar sweetened soft drinks beverages.

Prevalence of obesity was compared between lifestyle patterns, adjusting for gender, age, educational level, geographical area and energy intake (Table 4). The prevalence odds ratio (POR) for obesity in men, 0.52 (IC 95% 0.29–0.92) allocated in the “*Mixed diet-physically active-low sedentary lifestyle pattern*” was significantly lower compared to those in the “*Poor diet-low physical activity-sedentary lifestyle pattern*”.

## 4. Discussion

In this cross-sectional study conducted in a random sample of Spanish adult population aged 18–64 years, four dietary patterns were characterized by factor analysis and subsequently used to identify three different lifestyle patterns based on the DPs, physical activities, sedentary time, and sleeping and smoking habits, using cluster analyses: a “*Mixed diet-physically active-low sedentary lifestyle pattern*”, a “*Not poor diet-low physical activity-low sedentary lifestyle pattern*” which included 63.3% of the sample, and a “*Poor diet-low physical activity-sedentary lifestyle pattern*”. A higher proportion of people aged 18–30 years was classified into the “*Poor diet-low physical activity-sedentary lifestyle pattern*”. The prevalence odds ratio for obesity in men allocated in the “*Mixed diet-physically active-low sedentary lifestyle pattern*” was significantly lower compared to those in the “*Poor diet-low physical activity-sedentary lifestyle pattern*”.

Analysis of dietary patterns has become an important approach in food consumption studies and nutritional epidemiology [11,27,28] Researchers report different methodological approaches and procedures to identify diet and lifestyle patterns, such as a priori defined patterns or data-driven, identified using principal components (PCA) [29], factor analysis [11,30], cluster analysis [31,32,33] and, more recently, hybrid methods [2]. Some pieces of research have identified lifestyle patterns associated to specific phenotypes and obesity in several population groups [34,35] and contributed to unravel the consequences that different diets may have on health [2,11,35,36,37,38].

In this study factor analysis was used to identify dietary patterns that were later used in cluster analysis. In the ANIBES study four DPs were singled out in adults. Similar DPs have been reported [37]. In Spain, in the context of the DORICA study, a pooled analysis of population-based cross-sectional surveys conducted between 1990 and 2000, using cluster analysis of three different DPs were characterized. A “*Meat rich DP*” with higher consumption of meat, cereals, and potatoes, an “*Unbalanced DP*” with higher consumption of milk and low consumption of vegetables and cereals, and a “*Mediterranean DP*” with higher consumption of vegetables, fruit, fish, and olive oil. The “*Unbalanced DP*” was associated with higher alcohol consumption, inadequate fruit and vegetable consumption and poor physical activity. This pattern was associated to higher prevalence odds ratio for metabolic syndrome. Conversely, the “*Mediterranean DP*” was associated with better fat intake quality profile and fiber intake [19].

More recently other authors have identified two DPs using factor analysis in Spanish adults in the context of Food4Me Project. A pattern with higher consumption of fast and processed foods, potatoes, red and white meats, processed cereals, and low consumption of fruit and vegetables, directly associated with BMI. A second DP showed higher consumption of eggs, fish, legumes, nuts, low-calorie beverages, oil, vegetables, and white meat [8]. 

Despite cross-sectional studies not allowing for causal inference, food patterns with higher energy density and low fiber content have been associated with higher obesity prevalence rates [2]. In longitudinal studies, such as the Baltimore Study, dietary patterns high in reduced-fat dairy, high fiber grains and cereals, vegetables and fruits, and low in meats, sugar-sweetened soft drinks, refined grains, and high fat dairy products, were significantly associated with lower weight gain, prospectively [39]. An energy-dense, high-saturated fat and low-fiber dietary pattern with high loadings of fast foods and snacks and low loadings of fruits and vegetables was shown to increase body weight, waist circumference, blood pressure, serum insulin, and lipid profile during a 10-year follow-up in severely obese Swedish adults [40]. In line with this, in adult Canadians a strong and consistent relation between an energy-dense, high-fat, low-fiber density dietary pattern and the risk of obesity was observed, significant in different population subgroups [2].

In the ANIBES study, 45.6% of male and 27.5% of female Spanish adults did not meet the recommendation of 150 min/week of moderate PA. In addition, 56.2% of male and 74% of female adults did not meet the recommendations for vigorous PA [13]. In this study we analyzed if these physical activity behaviors combined in a meaningful way with other energy balance-related behaviors, DPs, and lifestyles. We also used this procedure to identify dietary and lifestyle patterns in Spanish children and adolescents and identified two patterns: an “*Unhealthier lifestyle pattern*” a combination of low physical activity and poorer diet, and a “*Healthier lifestyle pattern*” which combined high physical activity, low sedentary behavior, longer sleep duration, and healthier diet.

Studies analyzing individual behaviors have found healthier eating habits, such as adequate fruit and vegetable consumption, to be more prevalent among women than in men [41], but adherence to physical activity recommendations were more prevalent in men than in women [42]. In line with the above, in this study “*Snack DP*” mean scores adjusted for age and energy intake were significantly higher in men than in women. Conversely, those of the “*Mediterranean DP*” were significantly higher in women. Men and women classified in the “*Mixed diet-physically active-low sedentary lifestyle pattern*” showed the highest mean scores for the “*Mediterranean DP*”. However, men in that lifestyle pattern showed the highest mean scores for the “*Snack DP*”, as well. The highest mean scores for the “*Dairy sweet DP*” were observed in men and women classified into the “*Poor diet-low physical activity-sedentary lifestyle pattern*”. 

In this study a significantly higher proportion of men was classified into the “*Mixed diet-physically active-low sedentary lifestyle pattern*” (71.9%), while significantly more women were classified into the “*Not poor diet-low physical activity-low sedentary lifestyle pattern*” (58.5%). Men classified in the “*Mixed diet-physically active-low sedentary lifestyle pattern*” were less likely to be obese (POR 0.52; 95% IC 0.29–0.93), compared to those in the “*Poor diet-low physical activity-sedentary lifestyle pattern*”. Cassidy et al. reported obese adults to be two to five times more likely to report an “unhealthy phenotype”, a combination of low physical activity, high TV viewing, and poor sleep duration compared to normal weight adults in the UK Biobank [43].

A significantly higher proportion of women classified into the “*Mixed diet-physically active-low sedentary lifestyle pattern*” had a high educational level (35.6%). Women with a low educational level were more likely to be obese compared to those highly educated. Clustering and co-occurrence of multiple risk behaviors has been reported to be more likely in men and women with lower education or intermediate educational level compared with those with higher education [44].

As shown in this study, individuals may follow a variety of unhealthy lifestyle behaviors which combine to favor weight gain, or mixtures of healthy and unhealthy practices. The findings in this study underline the importance of designing and implementing interventions that address multiple health risk habits, considering lifestyle patterns, clustering of risk behaviors, and associated determinants. It is recognized that interventions which target more than one risk behavior have the potential for greater health benefits, enhance health promotion opportunities and can contribute to reduce health care costs. Such an approach allows for more adequate tailoring of interventions to participants’ profiles and helps to address health inequalities, reinforcing policies to ensure interventions accessible to socially-disadvantaged groups. 

The ANIBES study has several strengths which include the careful design, protocol, and methodology used for dietary data collection and validated questionnaires used to collect information on physical activity which have shown good reliability and reproducibility. The study was conducted on a random population sample of the Spanish population aged 9–75 years. A limitation of this study is its cross-sectional design, which provides evidence for associations but not causal relationships. Measures of physical activity relied on self-reports and could be biased, although a careful multistep quality control procedure was implemented to minimize bias. In addition, we used factor analysis to identify DPs based on food consumption information collected by three-day food records and one 24-h recall, considering food groups to further collapse dietary intake data and avoid missing data from non-consumers of episodically consumed foods. Finally, both factor analysis and cluster analysis are procedures commonly used to identify DPs and analyze clustering of lifestyles. However, those procedures rely on several subjective decisions that may influence outcomes regarding the number and type of patterns and clusters identified.

## 5. Conclusions

Four dietary patterns were characterized in Spanish adults aged 18–64 years, one of them closer to the Mediterranean DP. These patterns were subsequently used to identify three different lifestyle patterns: a “*Mixed diet-physically active-low sedentary lifestyle pattern*”, a “*Not poor diet-low physical activity-low sedentary lifestyle pattern*” which included 63.3% of the sample, and a “*Poor diet-low physical activity-sedentary lifestyle pattern*”. A higher proportion of people aged 18–30 years was classified into the “*Poor diet-low physical activity-sedentary lifestyle pattern*”. The prevalence odds ratio for obesity in men allocated in the “*Mixed diet-physically active-low sedentary lifestyle pattern*” was significantly lower compared to those in the “*Poor diet-low physical activity-sedentary lifestyle pattern*”. Although prospective research on large population samples would be desirable to further analyze and track over time the lifestyle patterns and how they influence the development of overweight and obesity, these behavior patterns are helpful to identify specific issues in population subgroups and inform intervention strategies. Further research is needed on factors associated with lifestyle patterns to gain insight in the population subgroups at higher risk. The findings in this study underline the importance of designing and implementing interventions that address multiple health risk practices, considering lifestyle patterns and associated determinants.

## Figures and Tables

**Table 1:** Gender, age group, educational level, geographical area, and BMI status by lifestyle pattern.

Characteristics	Mixed Diet-Physically Active-Low Sedentary Lifestyle Pattern		Not Poor Diet-Low Physical Activity-Low Sedentary Lifestyle Pattern		Poor Diet-Low Physical Activity-Sedentary Lifestyle Pattern		All	χ^2^	*p*
	*n*	%	*n*	%	*n*	%	*n*		
All	210	13.0	1020	63.3	381	23.6	1611		
Gender									
Men	151	71.9	423	41.5	204	53.5	778	70.1	0.000
Women	59	28.1	597	58.5	177	46.5	833		
Age group									
18–30 years	61	29.0	213	20.9	139	36.5	413	60.3	0.000
31–49 years	110	52.4	487	47.7	183	48.0	780		
50–64 years	39	18.6	320	31.4	59	15.5	418		
Educational level								27.8	0.000
Primary or less	55	26.2	304	29.8	73	19.2	432		
Secondary	95	45.2	507	49.7	189	49.6	791		
Higher	60	28.6	209	20.5	119	31.2	388		
Geographical area									
North-northwest	36	17.1	166	16.3	74	19.4	276	4.7	0.577
Eastern-Mediterranean	70	33.3	350	34.3	127	33.3	547		
Center	48	22.9	233	22.8	96	25.2	377		
South	56	26.7	271	26.6	84	22.0	411		
BMI status									
Normal weight	88	41.9	385	37.7	177	46.5	650	17.3	0.002
Overweight	90	42.9	386	37.8	136	35.7	612		
Obese	32	15.2	249	24.4	68	17.8	349		

Pearson’s chi-square tests were used to investigate the differences in cluster distribution by gender, age group, education level, geographical area, and BMI status.

**Table 2:** Physical activity behaviors, sedentary, sleep time on weekdays, smoking behavior, and dietary pattern scores in the lifestyle patterns by gender *.

	Mixed Diet-Physically Active-Low Sedentary Lifestyle Pattern			Not Poor Diet-Low Physical Activity-Low Sedentary Lifestyle Pattern			Poor Diet-Low Physical Activity-Sedentary Lifestyle Pattern			*F*	*p*
	Mean	SD	Median	Mean	SD	Median	Mean	SD	Median		
Men	*n* = 151			*n* = 423			*n* = 204				
“*Traditional DP*” *score*	0.04	1.03	0.00	0.05	1.04	0.00	−0.03	0.95	0.00	1.2	0.314
“*Mediterranean DP*” *score*	0.25	1.35	0.06	−0.17	1.00	−0.23	−0.12	0.96	−0.09	8.0	0.000
“*Snack DP*” *score*	0.66	1.29	0.52	0.14	0.95	0.07	0.46	1.14	0.31	9.9	0.000
“*Dairy-sweet DP*” *score*	−0.05	0.99	−0.11	−0.49	0.61	−0.51	1.03	1.21	0.88	126.9	0.000
Walking (min/week)	447.8	434.4	240.0	284.7	291.7	210.0	240.7	274.9	150.0	20.3	0.000
Moderate PA (min/week)	478.4	426.9	360.0	321.1	360.1	180.0	174.1	234.8	112.5	32.8	0.000
Vigorous PA (min/week)	706.4	291.7	720.0	81.8	127.3	0.0	102.3	155.4	0.0	734.1	0.000
Sedentary time (h/day)	3.6	2.0	3.0	4.4	2.3	4.0	7.1	3.7	6.4	96.8	0.000
Sleeping (h/day)	6.6	2.3	7.0	7.1	1.9	7.5	7.0	2.0	7.0	3.9	0.021
Smoking (cig/day)	4.3	6.9	0.0	6.4	8.7	0.0	3.1	6.0	0.0	10.2	0.000
Women	*n* = 59			*n* = 597			*n* = 177				
“*Traditional DP*” *score*	−0.30	1.02	−0.20	0.02	0.96	−0.02	−0.12	1.00	−0.10	7.2	0.001
“*Mediterranean DP*” *score*	0.33	1.06	0.45	0.03	0.89	0.05	0.09	0.92	0.09	7.2	0.001
“*Snack DP*” *score*	−0.20	0.94	−0.37	−0.38	0.75	−0.49	−0.12	0.82	−0.29	7.2	0.001
*Dairy-sweet DP’ score*	0.03	0.74	−0.02	−0.33	0.60	−0.36	1.16	0.89	1.09	203.0	0.000
Walking (min/week)	528.6	364.1	420.0	267.0	270.2	180.0	247.5	263.0	180.0	25.6	0.000
Moderate PA (min/week)	740.3	399.6	750.0	545.8	442.9	420.0	316.9	332.2	210.0	25.2	0.000
Vigorous PA (min/week)	692.0	325.3	630.0	45.9	92.2	0.0	57.8	120.2	0.0	685.4	0.000
Sedentary time (h/day)	3.6	2.1	3.0	4.0	2.2	4.0	6.6	4.1	6.0	60.5	0.000
Sleeping (h/day)	6.2	2.5	7.0	7.1	1.9	7.5	7.0	2.2	7.5	6.3	0.002
Smoking (cig/day)	2.5	5.6	0.0	3.9	7.0	0.0	2.3	5.2	0.0	4.5	0.011

* Physical activities, sedentary time, sleep, and smoking compared by ANOVA considering age group. General linear models adjusted for age and energy intake to compare DP scores.

**Table 3:** Consumption of selected food groups and beverages by lifestyle pattern in men and women.

	**Mixed Diet-Physically Active-Low Sedentary Lifestyle Pattern**			**Not Poor Diet-Low Physical Activity-Low Sedentary Lifestyle Pattern**			**Poor Diet-Low Physical Activity-Sedentary Lifestyle Pattern**			***F***	***p***
	**Mean**	**SD**	**Median**	**Mean**	**SD**	**Median**	**Mean**	**SD**	**Median**		
***Men* (*n* = 781)**	**(*n* = 151)**			**(*n* = 423)**			**(*n* = 204)**				
Vegetables (g/day)	184.9	110.9	165.4	185.1	112.4	162.5	178.2	97.2	165.0	0.98	0.374
Fruit (g/day)	183.1	231.0	136.7	145.0	172.7	97.5	139.5	144.0	103.9	3.24	0.040
Legumes (g/day)	16.7	23.1	7.5	16.2	19.1	10.5	13.6	18.2	7.1	2.19	0.113
Meat (g/day)	127.2	92.3	111.7	109.8	75.3	95.8	124.9	77.4	116.3	0.56	0.573
Processed and cold meats (g/day)	55.8	46.6	44.3	42.5	36.0	34.2	50.3	39.1	45.9	2.46	0.086
Fish (g/day)	73.6	90.8	47.7	62.5	66.9	39.3	55.8	57.1	35.4	3.01	0.050
Eggs (g/day)	40.8	46.4	31.3	32.5	33.4	21.3	28.3	30.4	20.0	9.58	0.000
Milk (mL/day)	155.5	122.9	139.7	125.6	100.7	115.0	267.5	178.5	249.4	60.42	0.000
Cheese (g/day)	25.4	41.4	15.2	15.8	20.0	10.0	19.2	22.2	12.9	4.39	0.013
Yoghourt (g/day)	62.3	74.5	41.7	42.3	64.2	0.0	46.2	62.0	20.8	3.62	0.027
Pasta (g/day)	22.6	27.5	12.5	16.2	20.0	11.7	17.7	20.2	11.7	3.08	0.047
Bread (g/day)	94.4	57.4	83.3	83.6	44.6	80.0	97.5	58.2	85.0	1.15	0.318
Cakes and pastry (g/day)	30.3	36.1	16.7	21.1	25.7	11.7	57.8	46.3	50.2	44.80	0.000
Sugar and sweets (g/day)	15.0	15.4	10.0	10.0	9.8	7.5	24.7	18.5	21.8	43.23	0.000
Pre-cooked foods (g/day)	73.0	83.0	50.0	76.3	86.9	45.8	80.0	91.3	46.3	3.45	0.032
Savory snacks (g/day)	6.1	12.1	0.0	4.7	10.1	0.0	7.5	14.3	0.0	0.51	0.603
Olive oil (mL/day)	20.0	8.9	20.2	18.0	8.8	16.7	17.3	7.5	18.0	6.40	0.002
Juices (mL/day)	71.0	123.9	0.0	40.0	79.8	0.0	88.6	175.4	0.0	1.51	0.221
Sugar sweetened soft drinks (mL/day)	104.2	151.5	41.7	97.7	186.7	0.0	127.2	192.2	47.5	2.44	0.088
Water (mL/day)	843.4	647.7	695.8	638.0	537.2	513.3	757.4	582.2	685.0	3.62	0.027
Alcoholic beverages (mL/day)	186.1	259.4	71.7	176.4	241.2	58.3	102.7	181.9	0.0	14.33	0.000
Low alcohol content bevs (mL/day)	1.6	5.7	0.0	2.8	11.6	0.0	3.4	19.4	0.0	2.95	0.05
High alcohol content bevs (mL/day)	184.5	257.6	71.7	173.6	238.8	55.8	99.3	177.3	0.0	14.31	0.00
	**Mixed Diet-Physically Active-Low Sedentary Lifestyle Pattern**			**Not Poor Diet-Low Physical Activity-Low Sedentary Lifestyle Pattern**			**Poor Diet-Low Physical Activity-Sedentary Lifestyle Pattern**			***F***	***p***
	**Mean**	**SD**	**Median**	**Mean**	**SD**	**Median**	**Mean**	**SD**	**Median**		
***Women* (*n =* 833)**	***n* = 59**			***n* = 597**			***n* = 177**				
Vegetables (g/day)	168.5	90.6	156.2	195.3	115.3	168.8	174.4	110.4	162.6	5.197	0.006
Fruit (g/day)	186.5	175.6	152.5	162.4	173.0	111.5	143.8	147.0	110.0	0.929	0.395
Legumes (g/day)	10.9	13.8	5.0	14.7	18.7	10.0	13.6	24.2	3.3	1.433	0.239
Meat (g/day)	78.8	63.1	66.7	90.9	63.4	82.5	101.6	70.1	89.2	2.054	0.129
Processed and cold meats (g/day)	37.1	31.5	29.7	33.5	30.1	26.7	37.5	32.2	29.2	1.582	0.206
Fish (g/day)	55.4	50.4	45.0	59.8	62.9	40.0	58.1	65.9	38.3	0.142	0.868
Eggs (g/day)	25.9	26.3	21.3	25.0	24.7	20.7	24.5	24.2	19.5	1.497	0.224
Milk (mL/day)	175.0	104.7	175.0	148.2	103.1	141.7	271.2	138.3	255.0	72.001	0.000
Cheese (g/day)	20.1	20.5	12.5	15.1	17.1	10.0	18.7	21.3	13.3	1.289	0.276
Yoghourt (g/day)	53.9	56.7	30.8	43.7	57.2	20.8	51.4	58.7	22.5	2.550	0.079
Pasta (g/day)	18.7	20.5	11.7	14.5	19.1	8.3	15.4	19.7	10.8	1.768	0.171
Bread (g/day)	64.8	38.4	56.7	65.7	39.6	60.0	66.7	33.7	64.2	11.989	0.000
Cakes and pastry (g/day)	31.6	37.1	20.7	20.9	22.6	15.0	56.3	40.9	50.0	54.345	0.000
Sugar and sweets (g/day)	15.6	12.4	13.7	11.3	11.3	8.2	30.2	25.0	25.3	52.816	0.000
Pre-cooked foods (g/day)	63.1	77.6	41.7	61.1	71.6	41.7	58.0	61.5	41.7	4.561	0.011
Savory snacks (g/day)	5.9	9.6	0.0	3.4	7.4	0.0	9.0	14.8	2.0	7.045	0.001
Olive oil (mL/day)	17.2	8.2	15.8	17.9	7.8	17.3	17.3	8.5	17.0	6.580	0.001
Juices (mL/day)	51.0	80.5	0.0	32.5	60.0	0.0	61.3	86.8	13.3	3.348	0.036
Sugar sweetened soft drinks (mL/day)	60.5	130.2	0.0	77.9	149.7	0.0	96.4	159.7	33.3	3.543	0.029
Water (mL/day)	764.2	563.1	658.3	649.0	474.2	550.0	753.6	560.2	641.7	4.689	0.009
Alcoholic beverages (mL/day)	84.4	132.5	30.0	68.5	141.6	0.0	47.0	100.4	0.0	8.842	0.000
Low alcohol content bevs (mL/day)	1.1	4.1	0.0	1.4	10.5	0.0	1.6	7.3	0.0	1.518	0.220
High alcohol content bevs (mL/day)	83.3	131.2	30.0	67.2	140.5	0.0	45.5	97.8	0.0	8.516	0.000

General linear models were used to estimate multivariate means for food consumption across lifestyle patterns adjusted for age and energy intake.

**Table 4:** Prevalence odds ratios (POR) of obesity in men and women, according to age group, level of education, geographical area, and lifestyle pattern.

	Men				Women			
	POR	95% C.I.POR		*p*	POR	95% C.I.POR		*p*
		Lower	Upper			Lower	Upper	
Age group								
50–64 years				0.000				0.000
18–30 years	0.29	0.17	0.48	0.000	0.29	0.16	0.52	0.000
31–49 years	0.61	0.42	0.89	0.011	0.52	0.35	0.78	0.002
Level of education								
High				0.293				0.000
Primary or less	1.41	0.87	2.28	0.162	3.11	1.74	5.58	0.000
Secondary	1.08	0.69	1.69	0.730	1.85	1.06	3.24	0.031
Geographical area								
South				0.017				0.704
North-northwest	1.13	0.65	1.99	0.658	0.83	0.45	1.50	0.532
Eastern-Mediterranean	1.99	1.25	3.16	0.003	1.11	0.70	1.76	0.672
Center	1.45	0.88	2.40	0.141	0.88	0.51	1.52	0.649
Lifestyle pattern								
Poor diet-low physical activity-sedentary lifestyle pattern				0.058				0.648
Mixed diet-physically active-low sedentary lifestyle pattern	0.52	0.29	0.93	0.027	1.16	0.48	2.80	0.738
Not poor diet-low physical activity-low sedentary lifestyle pattern	0.92	0.59	1.44	0.726	1.30	0.75	2.25	0.358

Note: Binary logistic regression models adjusted for age group, educational level, geographical area, and energy intake.

## Supplementary Materials

The following are available online at www.mdpi.com/2072-6643/9/6/606/s1, Table S1: Food groups and subgroups in the ANIBES Study, Table S2: Characteristics of the sample analyzed, Figure S1: Final cluster center scores for lifestyle patterns identified in Spanish adults aged 18–64 years in the ANIBES study.
