# Supplementary material for: Lifestyle Patterns and Weight Status in Spanish Adults: The ANIBES Study
Source: Nutrients. 2017 Jun 14;9(6):606. doi: 10.3390/nu9060606 (PMC5490585; doi:10.3390/nu9060606)
Supplement: Supplementary file 1 [file nutrients-09-00606-s001.zip › nutrients-192834-supplementary.pdf]

Supplementary Table S1 Food groups[a] and subgroups in the ANIBES Study

**Grains**
Grains and flours
Bread
Breakfast cereals and cereal bars
Pasta
Bakery and pastry
**Vegetables**
**Fruits**
**Oils and fats**
Olive oil
Other oils
Butter, margarine and shortening
**Milk and dairy products**
Milks
Cheeses
Yogurt and fermented milk
Other dairy products
**Fish and Shellfish**
**Meat and meat products**
Meat
Sausages and other meat products
Viscera and spoils
**Eggs**
**Pulses**
**Sugars and sweets**
Sugar
Chocolates
Jams and other
Other sweets
**Appetizers**
**Ready-to-eat-meals**
**Sauces and condiments**
**Non-alcoholic beverage**
Water
Coffee and infusions
Sugar soft drinks
Non-sweetened soft drinks
Sports Drinks
Energy drinks
Juices and nectars
Other drinks
**Alcoholic beverages**
Low alcohol content beverages
High alcohol content beverages
**Supplements and meal replacement**

[a] Olive oil: olive oil and virgin olive oil; Vegetables: All vegetables, including root vegetables and potatoes; Fruit: all fresh, canned, and dried fruits, including nuts and seeds; Pulses: green and dried pulses; Bread: all types of bread, including whole grain; Baked goods: Biscuits, sweet breads, and cakes; Sugar and sugary products: sugar, sweets, chocolates, confectionery, jam, marmalade, turron; Milk: all types of milk; Cheese: all cheeses; Yoghurt and fermented milk: all yoghurt and fermented milk products; Meat and meat products: all meat and meat products; Cold and processed meats: sausages and other cold meats; Sugared soft drinks: any flavor sugared soft drinks and sodas; Juices: juices and nectars; Sauces and dressings: sauces, dressings, condiments, and spices.

Supplementary Table S2.- Characteristics of the sample analyzed

	Men		Women		All		χ^2	P
	n	%	n	%	n	%		
Age group								
18- 30 y.	207	26.50	207	24.76	414	25.60	0.82	0.664
31-49 y	378	48.40	407	48.68	785	48.55		
50-64 y	196	25.10	222	26.56	418	25.85		
Total	781	100	836	100	1617	100		
Educational level								
Primary of less	207	26.50	226	27.03	433	26.78	0.31	0.856
Secondary	389	49.81	405	48.44	794	49.10		
Higher	185	23.69	205	24.52	390	24.12		
Total	781	100	836	100	1617	100		
Geographical area								
North-northwest	136	17.4	143	17.1	279	17.3	3.05	0.385
Eastern-Mediterranean	255	32.7	292	34.9	547	33.8		
Center	197	25.2	182	21.8	379	23.4		
South	193	24.7	219	26.2	412	25.5		
BMI status								
Normal weight	245	31.37	408	48.80	653	40.38	51.08	0.000
Overweight	339	43.41	275	32.89	614	37.97		
Obese	197	25.22	153	18.30	350	21.65		
Total	781	100	836	100	1617	100		

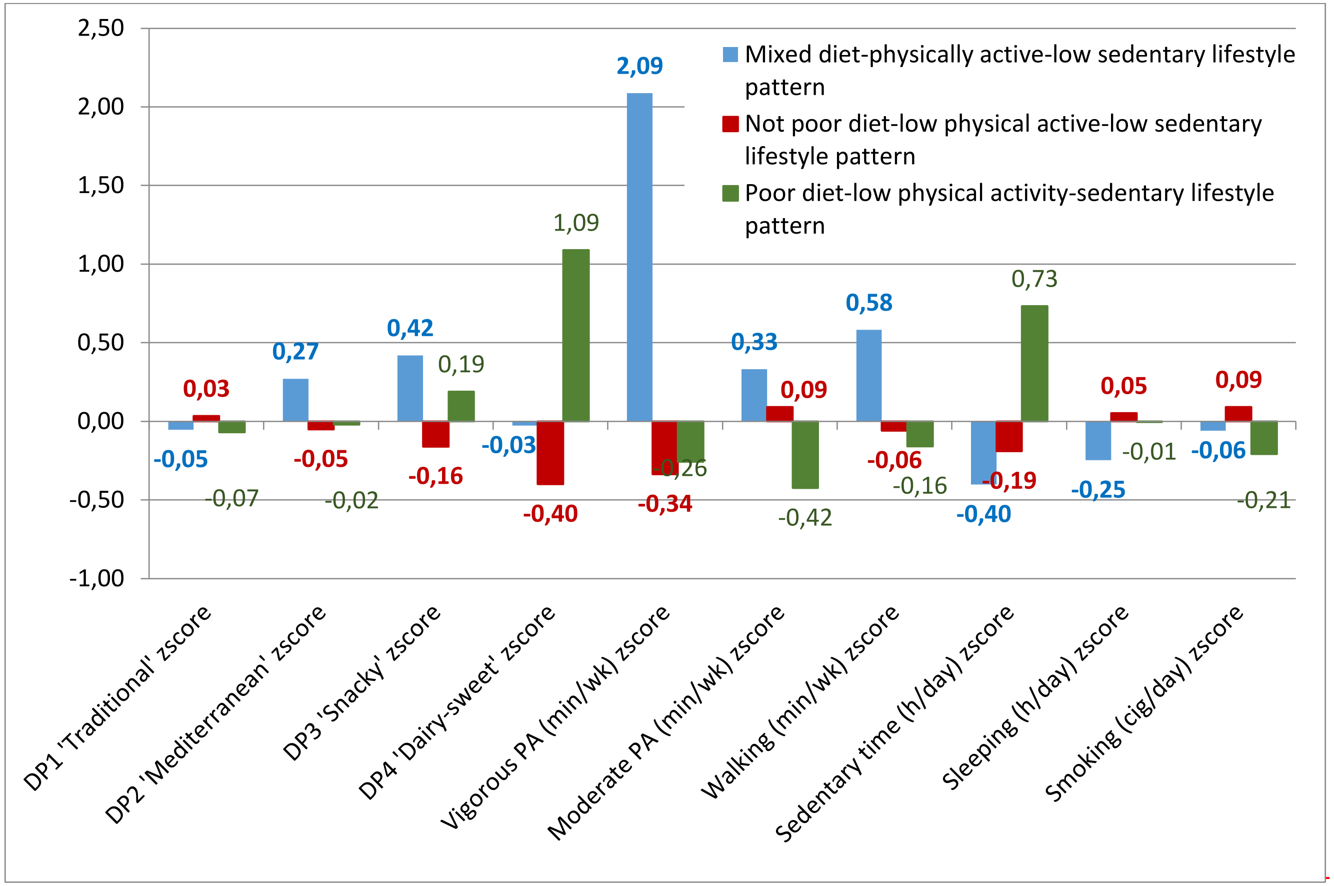

Mixed diet-physically active-low sedentary lifestyle pattern
Not poor diet-low physical active-low sedentary lifestyle pattern
Poor diet-low physical activity-sedentary lifestyle pattern
2,50
2,00
1,50
1,00
0,50
0,00
-0,50
-1,00
DP1 'Traditional' zscore
DP2 'Mediterranean' zscore
DP3 'Snacky' zscore
DP4 'Dairy-sweet' zscore
Vigorous PA (min/wk) zscore
Moderate PA (min/wk) zscore
Walking (min/wk) zscore
Sedentary time (h/day) zscore
Sleeping (h/day) zscore
Smoking (cig/day) zscore
-0,05
0,03
-0,07
0,27
-0,05
-0,02
0,42
-0,16
0,19
-0,03
-0,40
1,09
2,09
-0,34
-0,26
0,33
0,09
-0,42
0,58
-0,06
-0,16
-0,40
-0,19
0,73
-0,25
0,05
-0,01
-0,06
0,09
-0,21

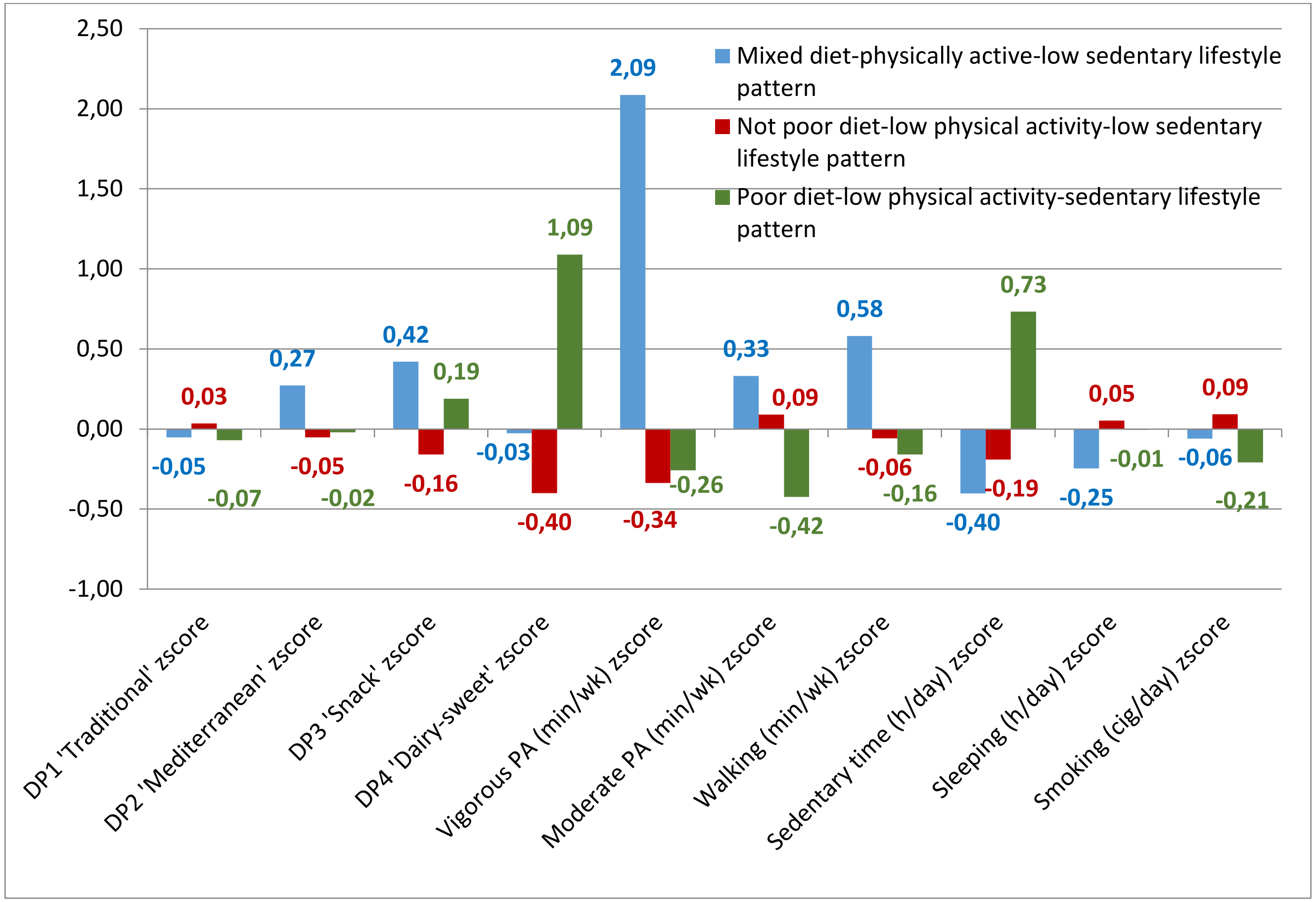

Figure S1.- Final cluster center scores for Lifestyle patterns identified in Spanish adults aged 18-64y in the ANIBES study
